# Not Just a Sum? Identifying Different Types of Interplay between Constituents in Combined Interventions

**DOI:** 10.1371/journal.pone.0125334

**Published:** 2015-05-12

**Authors:** Katrijn Van Deun, Lieven Thorrez, Robert A. van den Berg, Age K. Smilde, Iven Van Mechelen

**Affiliations:** 1 Research Group of Quantitative Psychology and Individual Differences, Faculty of Psychology and Educational Sciences, KU Leuven, Belgium; 2 KU Leuven—University of Leuven, Department of Development and Regeneration @ Kulak, Kortrijk, Belgium; 3 GlaxoSmithKline Vaccines, Rixensart, Belgium; 4 Biosystems data analysis, Swammerdam Institute for Life Sciences, University of Amsterdam, Amsterdam, The Netherlands; 5 Methodology and Statistics, Tilburg University, Tilburg, The Netherlands; Queen's University Belfast, UNITED KINGDOM

## Abstract

**Motivation:**

Experiments in which the effect of combined manipulations is compared with the effects of their pure constituents have received a great deal of attention. Examples include the study of combination therapies and the comparison of double and single knockout model organisms. Often the effect of the combined manipulation is not a mere addition of the effects of its constituents, with quite different forms of interplay between the constituents being possible. Yet, a well-formalized taxonomy of possible forms of interplay is lacking, let alone a statistical methodology to test for their presence in empirical data.

**Results:**

Starting from a taxonomy of a broad range of forms of interplay between constituents of a combined manipulation, we propose a sound statistical hypothesis testing framework to test for the presence of each particular form of interplay. We illustrate the framework with analyses of public gene expression data on the combined treatment of dendritic cells with curdlan and GM-CSF and show that these lead to valuable insights into the mode of action of the constituent treatments and their combination.

**Availability and Implementation:**

R code implementing the statistical testing procedure for microarray gene expression data is available as supplementary material. The data are available from the Gene Expression Omnibus with accession number GSE32986.

## Introduction

An important theme in research on treatments, interventions, and other forms of manipulations, is the study of combined manipulations. Examples include the study of multidrug therapies and the study of double knockout model organisms. In such studies one typically investigates the effect of the combined manipulation and of its constituents on one or several outcomes of interest (e.g., outcomes at the phenotypic level like clinical effectiveness, or outcomes at the molecular level like mRNA transcription rates). In this paper, we focus on studies of combined manipulations with two constituents that are systematically included vs. excluded according to a 2x2 experimental design with outcomes at a molecular level. Examples of such studies include investigations into the combination of the adjuvants CpG and MF59 for enhanced vaccine efficacy [1], into the combination of the multi-kinase inhibitor sorafenib and the non-steroidal anti-inflammatory drug diclofenac in the treatment of melanoma [2], into the effect of the co-deletion of phosphatase and tensin homologue (PTEN) and suppressor of cytokine signalling 3 (SOCS3) on axon regeneration [3], and into the combined effects of a model air pollutant and oxidized 1-palmitoyl-2-arachidonyl-sn-glycero-3-phosphorylcholine on genome-wide gene expression [4].

A major research question in such combination studies pertains to the type of interplay between the constituents when they are combined. In this regard, different types of interplay have been distinguished in the literature [5,6,7]. One form of interplay that can be singled out at this point is synergism, which is used to describe situations in which the effect of the combined treatment exceeds the sum of the effects of its constituents. The possibility of synergistic effects is a major motivation for the use of drug combinations in the treatment of diseases that are difficult to treat otherwise, such as various forms of cancer, which are often characterized by multiple abnormalities that each may be targeted by a different treatment component [8,9]. Another form of interplay is of an emergent (sometimes also called coalistic) type [6]: No effect is seen for each of the constituents, unlike for the combined manipulation. This form of interplay could, for example, occur when the expression of a target gene requires that two transcription factors each need to bind, with each constituent intervention activating one transcription factor only [10].

So far, common approaches that have been used to analyze data of studies with a 2x2 design of combined interventions are based on pairwise comparisons between some of the four conditions of the design. For example, in transcriptomics, a typical approach is to compare each of the three treatment conditions with the control condition, and to subsequently look for overlap and differences in the three resulting lists of differentially expressed genes [1,11]. Yet, such analyses are not suitable to test for the presence of a particular form of interplay for the following reasons: (a) The forms of interplay correspond to patterns in the data that are based on a conjunctive combination of several comparisons between conditions. For example, synergy occurs if and only if it simultaneously holds that each of the two single constituent conditions differs from the control condition and that the effect of the combined manipulation is more than the addition of the effects of its constituents; hence, synergy involves a combination of three comparisons. (b) All patterns involve a comparison with aggregated effects of conditions. The synergistic pattern, for example, involves that the effect of the combined manipulation is compared with the sum of the effects of the two constituents.

The present paper aims at closing the methodological gap implied by the shortcomings of common approaches to the study of combined interventions mentioned above. For this purpose, we will first outline a taxonomy of a broad range of forms of interplay between the two constituents of a combined manipulation. Subsequently, we will propose a sound and tailor-made statistical methodology to test for the presence of each particular form of interplay in this taxonomy. As an illustration of the methodology, we will apply it to genomewide expression data obtained from the public domain.

## Methods

Different forms of interplay between two constituents have been described in the literature on combination therapies, including the research on dose-effect relations for multidrug combinations as investigated in pharmacodynamics; see, for example, the review papers [5,6,8,12]. However, so far a broad and well-formalized taxonomy of possible forms of interplay as these may be captured in experimental studies with fully factorial designs is lacking. This is even more the case for a taxonomy linked to a tailor-made statistical methodology to tell apart these forms on the basis of empirical data. Here, we offer such a well-formalized taxonomy within the setting of a 2x2 experimental design, along with a methodology with a firm statistical basis to test for the presence of each of the reported forms of interplay. In addition, we will also present an implementation of this methodology that is suitable for outcomes on a molecular level, namely gene expression microarray data.

### Taxonomy

A 2x2 experimental design is supposed to underlie the data with as factors two manipulations that are either present or absent. A tabular representation of this design is given in Table 1; A and B denote the conditions with only one manipulation, AB the condition with the combined manipulation, and C the control condition.

**Table 1:** The 2x2 experimental design for combining two manipulations.

		Manipulation 1	
		Absent	Present
**Manipulation 2**	**Present**	B	AB
	**Absent**	C	A

When conducting an experiment, interest is in the effect of the manipulations, that is, within the present context, in the difference in the target outcome between the conditions A, B, and AB on the one hand and the control condition C on the other hand. For the time being, we limit ourselves to the case that all effects are nonnegative (*μ*_A_ ≥ *μ*_C_, *μ*_B_ ≥ *μ*_C_, and *μ*_AB_ ≥ *μ*_C_ with *μ*_X_ denoting the expected outcome in condition *X*) and for which the combined manipulation is effective: *μ*_AB_> *μ*_C_. A critical and primary comparison in the construction of our taxonomy is the one between the effect of the combined manipulation and the sum of effects of the constituents: **(** *μ*_AB_-*μ*_C_) compared to (*μ*_A_-*μ*_C_)+ (*μ*_B_-*μ*_C_). In particular, the effect of the combined manipulation can be either larger, (approximately) equal, or smaller than the sum of effects of the constituents. Secondary comparisons pertain to the effectiveness of each of the constituents: They can be both effective, only one of them can be effective, or none of the two. This gives rise to 12 combinations, two of which are logically impossible (due to the assumption *μ*_AB_>*μ*_C_), thus yielding a taxonomy with 10 different forms of interplay. These are summarized in Table 2.

**Table 2:** Taxonomy of ten possible forms of interplay between a combined manipulation AB and its single constituents A and B.

	(*μ*_AB_-*μ*_C_) < (*μ*_A_-*μ*_C_) + (*μ*_B_-*μ*_C_)	(*μ*_AB_-*μ*_C_) ≈ (*μ*_A_-*μ*_C_) + (*μ*_B_-*μ*_C_)	(*μ*_AB_-*μ*_C_) > (*μ*_A_-*μ*_C_) + (*μ*_B_-*μ*_C_)
*μ*_A_ > *μ*_C_ and *μ*_B_ > *μ*_C_	ANTAGONISM	ADDITIVE	SYNERGISM
*μ*_B_ > *μ*_C_ and *μ*_A_ ≈ *μ*_C_	REDUCTIVE by A	REDUNDANCE of A	POTENTIATION by A
*μ*_A_ > *μ*_C_ and *μ*_B_ ≈ *μ*_C_	REDUCTIVE by B	REDUNDANCE of B	POTENTIATION by B
*μ*_A_ ≈ *μ*_C_ and *μ*_B_ ≈ *μ*_C_	(not possible)	(not possible)	EMERGENT

Focus is on the effects of the manipulations, that is, the difference with control condition C.

The additive, synergistic, antagonistic, and potentiation forms of interplay are best known (see, for example [5]). In the review by [6] the reductive and redundant forms of interplay have also been discussed. The form of interplay that we call emergent is mentioned with the label coalistic in the papers [6] and [12]; note that ‘emergent’ is a label borrowed from systems theory and the study of categories and concepts in philosophy and cognitive science [13].

Let us now consider more in detail the biological relevance of the different forms of interplay as distinguished in Table 2. Firstly, we focus on the forms with all single and combined manipulations being effective, that is, the additive, synergistic, and antagonistic forms of interplay (the first row in Table 2). An additive effect will be observed when the two constituents contribute, biologically speaking, in an independent way to the overall effect; this may be the case, for example, when the constituents act on the same target without reaching saturation, or when each constituent affects one out of two independent pathways. The synergistic or ‘more-than-additive’ effect is often of great interest because of its potential practical relevance; for example, it may allow to reduce the doses of the two constituents resulting in less toxicity or side effects. From its part, an antagonistic effect may be observed in case of saturation or of non-specific binding site conformations where one of the constituents locks the target for binding by products resulting from the operation of the other.

Secondly, forms of interplay where one or both of the constituents are ineffective, are potentiation, redundance, and the reductive and emergent forms of interplay (the second and third row of Table 2). Potentiation means that, whereas one of the manipulations does not yield an effect in itself, when combined with the other manipulation the effect of the latter is enhanced. As an example, [8] and [12] discuss the well-known case of amoxicilin and clavulanate to treat bacterial infections. Clavulanate in its own has no antibacterial properties; yet, as it inhibits the enzyme that leads to destruction of amoxicillin, the antibacterial effect of amoxicillin is strongly enhanced when administered together with clavulanate. The reductive (or, inhibitory) form of interplay from its part may also be of practical interest if this form of interplay would show up in a pathway that influences toxicity (while not affecting the pathways that influence the therapeutic effect); for example, cisplatin and procainamide may be combined in the treatment of cancer, with procainamide being used to reduce the hepatotoxicity of cisplatin. The emergent form of interplay is not often reported in multidrug studies, as these typically focus on active compounds. In knock-out experiments, however, cases have been reported where no effect is seen in the single knock-out models unlike in its double knock-out counterpart (see for example [14]). Finally, the redundant form of interplay may simply show up when one of the constituents is irrelevant for pathways targeted by the other.

So far, we limited ourselves to situations in which all effects were positive (or at least nonnegative). In the case of gene expression data this corresponds to up-regulation. Yet, for this kind of data it also makes sense to consider negative effects (down-regulation). An analogous taxonomy as described above could be used in this case. For example, a synergistic down-regulating effect means that *μ*_A_<*μ*_C_, and *μ*_B_< *μ*_C_, and (*μ*_AB_- *μ*_C_)<(*μ*_A_- *μ*_C_)+(*μ*_B_—*μ*_C_). (Note that *μ*_AB_< *μ*_C_ then logically follows, so the effect is down-regulating, indeed.)

### Statistical methodology to test for presence of form of interplay

Each of the forms of interplay as defined in the taxonomy summarized in Table 2, is defined by a set of inequality and (approximate) equality relations that have to hold simultaneously true. To cast the problem of identifying a particular form of interplay into a statistical framework, we translate it into a hypothesis testing problem in which each of the inequality and (approximate) equality relations is formalized as an alternative partial hypothesis H1*i* and the complement as a partial null hypothesis H0*i*. These partial hypotheses then are combined in the following way: The compound null hypothesis H0 is the union of H0*i* (across all *i*) and the compound alternative hypothesis H1 the intersection of H1*i* (across all *i*). In this way a test problem is obtained where the compound null hypothesis H0 that at least one of the relations is not true can be rejected against the compound alternative H1 that all inequalities and equivalences are true. It is important to note that this problem is different from a multiple testing setup in which an H0 that is an intersection of H0*i* is to be tested against a union of H1*i* (implying that H1 is ‘accepted’ when at least one of the partial null hypotheses is rejected).

To test each of the partial hypotheses we use either contrasts or equivalence testing depending on whether the partial hypothesis pertains to an inequality or an (approximate) equality relation. In particular, the presence of an inequality relation, for example, *μ*_A_> *μ*_C_, can be tested by testing H0*i*: *μ*_A_ = *μ*_C_ against H1*i*: *μ*_A_> *μ*_C_. One may note that this is equivalent to testing H0*i*: *μ*_A_- *μ*_C_ = 0 against H1*i*: *μ*_A_- *μ*_C_ >0, which comes down to a one-sided test of the contrast ψ = *μ*_A_-*μ*_C_. Regarding approximate or near-equality relations, for example A≈C, we can rely on a so-called “Two One Sided Tests” procedure (TOST; see for example [15,16]). This procedure starts from the working hypothesis that the difference in population means lies within some pre-defined tolerance interval [*ε*_lower_, *ε*_upper_], and implies a test of H0: *μ*_A_- *μ*_C_≤*ε*_lower_ *or μ*_A_- *μ*_C_≥*ε*_upper_ against H1: *μ*_A_- *μ*_C_>*ε*_lower_ *and μ*_A_- *μ*_C_< *ε*_upper_ (or, H1: *ε*_lower_< *μ*_A_- *μ*_C<_ *ε*_upper_) with *ε*_lower_<0 and *ε*_upper_>0. Note that this is again a situation of testing a union of H0*i* against an intersection of H1*i*. To test, for example, H0*i*: *μ*_A_- *μ*_C_≥*ε*_upper_ the following test statistic can be used,

$$U=\frac{\varepsilon_{upper}-\left({\bar{y}}_{A}-{\bar{y}}_{C}\right)}{SE\left({\bar{y}}_{A}-{\bar{y}}_{C}\right)}$$ (1)

with *ε*_upper_ >0 a pre-set tolerance limit and SE(*Y*) denoting the standard error of *Y*. H1*i*: *μ*_A_- *μ*_C_<*ε*_upper_ is accepted when *U* exceeds the critical value t_df,1-*α*_ with *α* a chosen significance level and *df* the degrees of freedom. Here, we will use symmetric equivalence intervals [*-ε*, *ε*] (this is, *ε*_lower_ = *-ε* and *ε*_upper_ = *ε*).

Finally, to test the compound hypothesis that all partial H0*i* are simultaneously rejected in favor of their alternatives, we rely on an intersection-union test [17,18]. As shown by [17], such an intersection-union test procedure implies that, when each partial hypothesis is tested at significance level α, this results in a significance level of at least α for the compound hypothesis.

### Implementation of the statistical methodology for gene expression data

To analyze the data while taking the 2x2 design of the study into account, the empirical Bayes procedure implemented in the R/Bioconductor package limma, version 3.18.13 [19,20] can be used. A property of this package, which is useful for our proposed methodology, is that it also allows to estimate contrasts and to assess their statistical significance. To test for near-equality, we implement the TOST procedure with statistic (1.1), making use of the moderated standard errors and degrees of freedom that are calculated by the empirical Bayes method. R code with an implementation of the procedure is included in the Supporting Information (S1 R code and S2 R code).

## Results

### Data structure and pre-processing

We illustrate our framework to identify particular forms of interplay between the constituents of a compound intervention with public microarray gene expression data accessible via the Gene Expression Omnibus with accession number GSE32986 [21]. These data were collected in a mouse study on the combined effect of the inflammatory growth factor GM-CSF and the dectin-1 agonist curdlan on dendritic cell maturation. Curdlan was produced as a water-insoluble polysaccharide by the soil bacterium, *Alcaligenes faecalis*. A tabular representation of the experimental design is given in Table 3: Bone marrow derived dendritic cells were either unstimulated or stimulated for 4 hours with 100μg/ml curdlan and/or with 5ng/ml GM-CSF. (The authors also included conditions with 1μg curdlan; they are used further in the manuscript.) For each condition, three independent samples were prepared and the extracted RNA was hybridized to the Affymetrix GeneChip Mouse Genome 430 2.0 arrays. The R (version 3.0.2) / Bioconductor (version 2.13) package affy (version 1.40.0; [22]) was used to obtain Robust Multichip Average (RMA) expression data; note that these are by default log2 transformed.

**Table 3:** Experimental design: Crossing of the treatments with curdlan (present/absent) and with GM-CSF (present/absent).

		GM-CSF	
		Absent	Present
curdlan	Present	CURDLAN	COMBINATION
	Absent	UNSTIMULATED	GM-CSF

### Forms of interplay for combining curdlan with GM-CSF in dendritic cells

#### Number of probesets displaying a particular form of interplay

We applied our method with the significance level set to. 05 and a tolerance interval for (approximate) equality equal to [-0.15, 0.15]. Note that this is a smaller interval than used in other applications of equivalence testing with genomewide expression data (see, e.g., [16]) but larger intervals would imply that some probesets would be classified in more than one form of interplay (which is possible if the standard error of the probeset is relatively small compared to the equivalence interval). The results are summarized in Table 4. In total, 1997 probesets display one of the forms of interplay listed in Table 3. The list of probesets together with the form of interplay they display is made available in the Supporting Information (S1 Table).

**Table 4:** Number of probe sets out of 45 101 found with a particular form of interplay between treatment with Curdlan (dose = 100μg or dose = 1μg), GM-CSF and their combination.

	Curdlan 100μg		Curdlan 1μg	
	UP	DOWN	UP	DOWN
**Synergistic**	49	49	38	1
**Additive**	22	130	13	23
**Antagonistic**	578	427	139	241
**Potentiation (by GM-CSF)**	34	58	9	1
**Redundance of GM-CSF**	63	105	17	13
**Reductive (by GM-CSF)**	10	1	1	0
**Potentiation (by Curdlan)**	7	23	32	50
**Redundance (of Curdlan)**	172	136	535	527
**Reductive (by Curdlan)**	64	21	16	24
**Emergent**	9	39	6	7

The significance level was set to. 05 and the tolerance limit for (approximate) equality to. 15.

Many probesets are found with a down-regulated pattern (compared to the unstimulated condition) although in the literature down-regulation is often neglected (e.g., [21] only discusses up-regulated genes). If curdlan and GM-CSF would, biologically speaking, operate in an independent way, only probesets in the redundant and additive category would be expected. However, our results show that many probesets display a form of interplay in which the constituents interact (with synergistic, emergent, potentiation, reductive, and antagonistic patterns). The predominant effect is antagonistic (1005 probesets out of 1997). This is not surprising as it may be explained by negative feedback loops in the pathways which generate a damped behavior, needed for homeostasis [23].

#### Pathway analysis

We used IPA (Ingenuity Systems, www.ingenuity.com) to find biological functions with a significant overrepresentation in the collections of probesets with the same form of interplay using a Benjamini—Hochberg corrected Fisher’s exact test with significance level. 05. For each form of interplay, one list of probesets was fed to IPA consisting of both the up- and down-regulated probesets. Significantly enriched terms were found for the following patterns: antagonistic, synergistic, potentiation by GM-CSF, reductive by GM-CSF, and redundance of curdlan. The enrichment results for potentiation and reductive suggest the presence of more biological functions in dendritic cells for which GM-CSF moderates the effect of curdlan rather than the other way around.

The results for potentiation by GM-CSF (see Table 5) suggest that GM-CSF does not activate quite a few pathways in itself but does so in presence of curdlan. Somewhat surprisingly, these also include the PI3K and MAPK/ERK signaling pathways, which are principal targets of GM-CSF. However, their activation by GM-CSF is known to depend both on the type of dendritic cell and on the condition of the cell (steady-state vs. an inflammatory condition), with in our case inflammation probably being triggered by curdlan [24]. Interestingly, [21] identified MAPK/ERK as one possible integration site of the signals produced by curdlan and GM-CSF; the present results shed more light on the nature of the integration.

**Table 5:** Canonical pathways with Benjamini-Hochberg p-value <. 05 for probesets showing potentiation or reduction by GM-CSF in the 2×2 experimental setup with 100μg Curdlan.

	Ingenuity Canonical Pathways	B-H *p*-value
**Potence by GMCSF**	RAR Activation	0.001
	CD28 Signaling in T Helper Cells	0.001
	iCOS-iCOSL Signaling in T Helper Cells	0.004
	Glucocorticoid Receptor Signaling	0.004
	B Cell Receptor Signaling	0.013
	FcγRIIB Signaling in B Lymphocytes	0.015
	fMLP Signaling in Neutrophils	0.019
	***PI3K Signaling in B Lymphocytes***	0.025
	Insulin Receptor Signaling	0.025
	Aryl Hydrocarbon Receptor Signaling	0.027
	PXR/RXR Activation	0.027
	Gαq Signaling	0.030
	Prolactin Signaling	0.032
	G-Protein Coupled Receptor Signaling	0.033
	Regulation of IL-2 Expression in Activated and Anergic T Lymphocytes	0.034
	Protein Kinase A Signaling	0.034
	Dopamine-DARPP32 Feedback in cAMP Signaling	0.034
	Xenobiotic Metabolism Signaling	0.035
	Role of NFAT in Regulation of the Immune Response	0.035
	G Beta Gamma Signaling	0.037
	HMGB1 Signaling	0.039
	IL-1 Signaling	0.039
	SAPK/JNK Signaling	0.040
	***ERK/MAPK Signaling***	0.040
	T Cell Receptor Signaling	0.040
	Telomerase Signaling	0.042
	Leukocyte Extravasation Signaling	0.042
	Fc Epsilon RI Signaling	0.046
	Androgen Signaling	0.049
	April Mediated Signaling	0.049
**Reductive by GMCSF**	Caveolar-mediated Endocytosis Signaling	0.038
**Redundance of Curdlan**	Protein Ubiquitination Pathway	0.000
	Aldosterone Signaling in Epithelial Cells	0.007
	EIF2 Signaling	0.012

*The canonical pathways in bold+ italic typeface are discussed in the manuscript.

The antagonistic, or less-than-additive associated pathways also include the PI3K and MAPK/ERK signaling module (see Table 6). The general PI3/AKT pathway integrates signals from multiple upstream elements and has multiple possible downstream effects such as regulation of pluripotency, energy storage, cell cycle progression, protein synthesis, and vasodilation; an antagonistic effect might take care to dampen certain parts of this broad PI3K/AKT pathway. In contrast, for potentiation by GM-CSF, the PI3K pathway contains a subset of elements from the PI3/AKT pathway, which is active for signaling in B lymphocytes. Thus, parts of the PI3/AKT pathway are damped whereas specific parts are potentiated.

**Table 6:** Canonical pathways with Benjamini-Hochberg *p*-value <. 05 for probesets showing antagonism or synergy in the 2×2 experimental setup with 100μg Curdlan.

	Ingenuity Canonical Pathways	B-H *p*-value
**Antagonistic**	G-Protein Coupled Receptor Signaling	0.000
	TREM1 Signaling	0.001
	***PI3K/AKT Signaling***	0.004
	***PTEN Signaling***	0.006
	Regulation of IL-2 Expression in Activated and Anergic T Lymphocytes	0.007
	MIF-mediated Glucocorticoid Regulation	0.007
	Granulocyte Adhesion and Diapedesis	0.008
	IL-17A Signaling in Fibroblasts	0.008
	Altered T Cell and B Cell Signaling in Rheumatoid Arthritis	0.008
	Gαi Signaling	0.014
	Role of RIG1-like Receptors in Antiviral Innate Immunity	0.019
	cAMP-mediated signaling	0.021
	LPS-stimulated MAPK Signaling	0.021
	Type I Diabetes Mellitus Signaling	0.029
	CD40 Signaling	0.029
	***Dendritic Cell Maturation***	0.032
	Role of PKR in Interferon Induction and Antiviral Response	0.042
	Role of IL-17A in Arthritis	0.043
	IL-6 Signaling	0.043
	MIF Regulation of Innate Immunity	0.043
	Acute Phase Response Signaling	0.045
	***ERK/MAPK Signaling***	0.045
**Synergistic**	Altered T Cell and B Cell Signaling in Rheumatoid Arthritis	0.000
	Graft-versus-Host Disease Signaling	0.001
	Differential Regulation of Cytokine Production in Macrophages and T Helper Cells by IL-17A and IL-17F	0.001
	Role of Cytokines in Mediating Communication between Immune Cells	0.001
	Differential Regulation of Cytokine Production in Intestinal Epithelial Cells by IL-17A and IL-17F	0.002
	Role of JAK family kinases in IL-6-type Cytokine Signaling	0.002
	IL-10 Signaling	0.002
	Hepatic Cholestasis	0.002
	Hepatic Fibrosis / Hepatic Stellate Cell Activation	0.002
	Communication between Innate and Adaptive Immune Cells	0.004
	Acute Phase Response Signaling	0.004
	PPAR Signaling	0.004
	Granulocyte Adhesion and Diapedesis	0.005
	***Dendritic Cell Maturation***	0.005
	Agranulocyte Adhesion and Diapedesis	0.005
	Role of IL-17F in Allergic Inflammatory Airway Diseases	0.005
	Sphingosine-1-phosphate Signaling	0.006
	IL-6 Signaling	0.007
	p38 MAPK Signaling	0.007
	LXR/RXR Activation	0.007
	RhoA Signaling	0.008
	TREM1 Signaling	0.015
	***NF-κB Signaling***	0.021
	FXR/RXR Activation	0.022
	IL-1 Signaling	0.026
	Role of Pattern Recognition Receptors in Recognition of Bacteria and Viruses	0.026
	Oncostatin M Signaling	0.034
	cAMP-mediated signaling	0.039
	Gα12/13 Signaling	0.042
	Gαi Signaling	0.045
	Phospholipase C Signaling	0.045

The canonical pathways in bold+ italic typeface are discussed in the manuscript.

To an important extent, dendritic cell maturation appears to be stimulated by curdlan and GM-CSF in an antagonistic manner. However, the dendritic cell maturation pathway was also significantly enriched in the collection of synergistic probesets, indicating that the combined use of GM-CSF and curdlan also boosts the maturation effect. In the antagonistic collection, the dendritic cell maturation affects CD40, CD80, and CD83, whereas the synergistic effect is observed for several interleukins. Interestingly, enrichment for IL-6 and IL17 signaling is seen, which may explain the specific effect on dendritic cell maturation. Indeed, IL-6 was described to regulate dendritic cell differentiation in vivo [25] and IL-17 promotes differentiation of dendritic cell progenitors [26]. Furthermore, in the synergistic list of canonical pathways found with Ingenuity (see Table 6), yet another important signaling module activated by GM-CSF shows up, namely NF-κ which is activated through the IκB kinase complex [24]. This pathway is also known to be activated by curdlan through the Syk and Raf-1 signaling pathways, where RAF1 occurs downstream of the MAPK pathway. Hence, a possible explanation for the observed synergy in nuclear translocation of different NF-kB subunits reported by [21], is the enhanced RAF activation by on the one hand curdlan in its own and on the other hand the MAPK signaling that results from a stimulation by both GM-CSF and curdlan. Note also the presence of the p38 MAPK signaling pathway in the synergistic group.

### Validation of the results using an additional experiment

The gene expression data made publicly available by [21] also include two conditions with 1μg curdlan, one using curdlan only and one using curdlan in conjunction with GM-CSF (5ng/ml) for stimulation. Together with the GM-CSF stimulated and the unstimulated cells, these yield a second 2×2 experimental setup that we will use for validation of the results obtained in the experiment with 100μg/ml curdlan. Note that the data of the two experimental setups are partially dependent as both contain the data from the GM-CSF stimulated and unstimulated cells. Yet, because of the dose variation in the curdlan constituent, the data have a clear structure of which we can take advantage to put forward several expectations (e.g., we can expect fewer patterns that build on an effect of curdlan in the low-dose setup).

In total, 1693 probesets display one of the forms of interplay; the numbers of probesets for each particular type of interplay are shown in the two rightmost columns of Table 4 (the list of probesets with affymetrix and gene identifiers is available from the Supporting Information, S2 Table). We will now compare these numbers with the corresponding numbers in the 2×2 experimental setup with the high-dose curdlan conditions (i.e., the second and third column of Table 4). As could be expected, the numbers of patterns which imply that curdlan on its own is effective (synergistic, additive, antagonistic, potentiation by GM-CSF, redundance of GM-CSF, and reductive by GM-CSF) are lower in the low-dose setup. One may further expect higher numbers of probesets for the patterns in which curdlan is ineffective, both on its own and in combination with GM-CSF (redundance of curdlan); for this type of interplay the numbers of probesets are considerably higher, indeed. For the patterns that are characterized by an absence of an effect of curdlan on its own but presence of an effect of curdlan when combined with GM-CSF (potentiation by curdlan, reductive by curdlan, and emergent) both an increase and a decrease of the numbers of probesets could be plausible. Here, we observe an increase of the number of probesets for potentiation and, a decrease for the emergent and reductive pattern.

For each collection of probesets with the same form of interplay that resulted from the low-dose curdlan setup, too, we ran ingenuity pathway analyses to find biological functions with a significant overrepresentation, using a Benjamini—Hochberg corrected Fisher’s exact test with significance level. 05. Significantly enriched terms were found for the synergistic, redundance of curdlan, and potentiation by curdlan patterns; see Table 7. For the former two categories, enriched terms were also found with the high dose of curdlan and these are mainly the same terms. For example, even in the low-dose curdlan setup, enrichment of the NF-kB, dendritic cell maturation, and p38 MAPK signaling pathways are confirmed for the synergistic probesets, which suggests conservation in the low-dose setup of the functional synergistic effects earlier found in the high-dose setup. To check that the similarity in annotation can be attributed to the overlap in genes between the two collections of probesets (one for the low-dose and one for the high-dose setup), we also performed a pathway analysis on the set of genes that are synergistic in both 2x2 experiments. The resulting functional annotation indeed recovers the shared terms (see S3 Table).

**Table 7:** Additional experiment with curdlan 1μg/ml: Canonical pathways with Benjamini-Hochberg *p*-value <. 05.

	Ingenuity Canonical Pathways	B-H *p*-value
**Synergistic**	Altered T Cell and B Cell Signaling in Rheumatoid Arthritis	0,00
	NF-κB Signaling	0,00
	Communication between Innate and Adaptive Immune Cells	0,00
	Hepatic Fibrosis / Hepatic Stellate Cell Activation	0,00
	Role of Pattern Recognition Receptors in Recognition of Bacteria and Viruses	0,00
	Graft-versus-Host Disease Signaling	0,00
	Acute Phase Response Signaling	0,00
	Granulocyte Adhesion and Diapedesis	0,00
	Dendritic Cell Maturation	0,00
	Agranulocyte Adhesion and Diapedesis	0,00
	Toll-like Receptor Signaling	0,00
	TREM1 Signaling	0,00
	Crosstalk between Dendritic Cells and Natural Killer Cells	0,00
	Differential Regulation of Cytokine Production in Intestinal Epithelial Cells by IL-17A and IL-17F	0,00
	HMGB1 Signaling	0,00
	B Cell Development	0,01
	Hepatic Cholestasis	0,01
	Autoimmune Thyroid Disease Signaling	0,01
	Role of Cytokines in Mediating Communication between Immune Cells	0,01
	CD40 Signaling	0,01
	IL-10 Signaling	0,01
	T Helper Cell Differentiation	0,01
	Regulation of IL-2 Expression in Activated and Anergic T Lymphocytes	0,02
	Allograft Rejection Signaling	0,02
	PPAR Signaling	0,02
	iCOS-iCOSL Signaling in T Helper Cells	0,03
	Type I Diabetes Mellitus Signaling	0,03
	Airway Pathology in Chronic Obstructive Pulmonary Disease	0,03
	IL-6 Signaling	0,03
	p38 MAPK Signaling	0,03
	CD28 Signaling in T Helper Cells	0,03
	PKCθ Signaling in T Lymphocytes	0,03
	LXR/RXR Activation	0,03
	FXR/RXR Activation	0,03
	PI3K Signaling in B Lymphocytes	0,03
	Aryl Hydrocarbon Receptor Signaling	0,03
	Role of IL-17A in Psoriasis	0,03
	Differential Regulation of Cytokine Production in Macrophages and T Helper Cells by IL-17A and IL-17F	0,04
	NRF2-mediated Oxidative Stress Response	0,05
**Redundance of Curdlan**	Protein Ubiquitination Pathway	0,00
	Regulation of eIF4 and p70S6K Signaling	0,00
	Androgen Signaling	0,02
	Estrogen Receptor Signaling	0,02
	EIF2 Signaling	0,02
	Hypoxia Signaling in the Cardiovascular System	0,02
**Potence by Curdlan**	RANK Signaling in Osteoclasts	0,03
	April Mediated Signaling	0,03
	B Cell Activating Factor Signaling	0,03
	TNFR1 Signaling	0,04

## Discussion and Conclusion

The study of combined manipulations is of great interest, for example, for pharmaceutical applications, given the observed successes with multi-drug treatments (see, for example, [12]). Very often interactions take place between the constituents of combined interventions and focusing on such interactions may be of primary importance to understand the mode of action of the constituents and their combination. Here, we offered a well-defined taxonomy of different forms the interplay between two constituents can take, along with a tailor-made statistical methodology to test for their presence. Importantly, the conceptual framework and the associated statistical methodology have a sound theoretical basis. To further show how they can be used in practice, we (1) provided an implementation of this methodology for gene expression microarray data, and (2) illustrated the methodology with an analysis of publicly available gene expression data on the combined treatment of dendritic cells with curdlan and GM-CSF. The study of the pathways obtained from enrichment analyses of lists of genes that display particular forms of interplay may lead to valuable insights into the mode of action of curdlan and GM-CSF and their combination. A proper validation of the biological results should be based on further experiments and the collection of new data, which is beyond the scope of the present methodological paper. Yet, we already found some encouraging support in the results of a 2×2 experimental setup that included two new low-dose curdlan conditions.

The approach proposed in the present paper could be significantly extended in several respects. First, we focused in this paper on studies with a 2×2 experimental design. However, various ways could be considered to extend the proposed taxonomy and associated statistical methodology to *K*×*K’* designs (with *K* and/or K’ >2); this would, for example, be most relevant for scenarios in which the two constituent interventions could be delivered with more than two doses (with absence of a constituent intervention corresponding to a zero dose condition). Possible ways of extension at this point may include regression-type approaches to the *K*×*K’* dataset as a whole (with the two dose variables and their product acting as predictors in the regression model), with an intersection-union test-based methodology focused on the three regression weights. Still another way of extension could be to apply the framework and methodology proposed in the present paper to several 2×2 parts of the *K*×*K’* data set (which would allow the researcher to investigate whether the form of interplay between the two constituent interventions is constant across the entire dose range). Second, in our taxonomy we limited ourselves to forms of interplay where the effects of the constituents and the combined manipulation were all either nonnegative or nonpositive, and where the effect of the combined manipulation was nonzero. This implies that cases in which a gene is up-regulated by one constituent and down-regulated by the other were not considered; moreover the same holds for cases in which at least one of the constituents is effective whereas the combined intervention is not. In some situations, however, such patterns could also be of interest. Examples may include the case in which the two constituent interventions imply some toxicity effect, whereas the combined intervention does not. Fortunately, the methodology proposed in the present paper can be easily extended to detect such forms of interplay.

In conclusion, the taxonomy and methodology proposed in the present paper constitute a sound and powerful tool to study the form of the interplay between the constituents of combined interventions. Moreover, the conceptual framework and associated methodology are versatile, in that they are applicable to a broad range of intervention types and types of outcome, and that they can be readily extended in several directions.

## Supporting Information

S1 R code R code used to find the forms of interplay: Experimental setup with a dose of 100μg/ml for curdlan. (R)

S2 R code R code used to find the forms of interplay: Experimental setup with a dose of 1μg/ml for curdlan. (R)

S1 Table Probeset identifiers, official gene symbols, statistics, and type of pattern for probesets displaying a particular form of interplay: Experimental setup with a dose of 100μg/ml for curdlan. (XLS)

S2 Table Probeset identifiers, official gene symbols, statistics, and type of pattern for probesets displaying a particular form of interplay Experimental setup with a dose of 1μg/ml for curdlan. (XLS)

S3 Table Ingenuity canonical pathways obtained for the list of genes displaying a synergistic pattern both in the 2x2 setup with 1μg and 100μg of curdlan. (XLS)

S1 Targets file Text file that matches the CEL file names with their experimental condition. (TXT)
